# Ammonia Oxidizing Bacteria Community Dynamics in a Pilot-Scale Wastewater Treatment Plant

**DOI:** 10.1371/journal.pone.0036272

**Published:** 2012-04-27

**Authors:** Xiaohui Wang, Xianghua Wen, Yu Xia, Ma Hu, Fang Zhao, Kun Ding

**Affiliations:** Environmental Simulation and Pollution Control State Key Joint Laboratory, School of Environment, Tsinghua University, Beijing, China; INRIA Sophia Antipolis – Méditerranée, France

## Abstract

**Background:**

Chemoautotrophic ammonia oxidizing bacteria (AOB) have the metabolic ability to oxidize ammonia to nitrite aerobically. This metabolic feature has been widely used, in combination with denitrification, to remove nitrogen from wastewater in wastewater treatment plants (WWTPs). However, the relative influence of specific deterministic environmental factors to AOB community dynamics in WWTP is uncertain. The ecological principles underlying AOB community dynamics and nitrification stability and how they are related are also poorly understood.

**Methodology/Principal Findings:**

The community dynamics of ammonia oxidizing bacteria (AOB) in a pilot-scale WWTP were monitored over a one-year period by Terminal Restriction Fragment Length Polymorphism (T-RFLP). During the study period, the effluent ammonia concentrations were almost below 2 mg/L, except for the first 60 days, indicting stable nitrification. T-RFLP results showed that, during the test period with stable nitrification, the AOB community structures were not stable, and the average change rate (every 15 days) of AOB community structures was 10%±8%. The correlations between T-RFLP profiles and 10 operational and environmental parameters were tested by Canonical Correlation Analysis (CCA) and Mantel test. The results indicated that the dynamics of AOB community correlated most strongly with Dissolved Oxygen (DO), effluent ammonia, effluent Biochemical Oxygen Demand (BOD) and temperature.

**Conclusions/Significance:**

This study suggests that nitrification stability is not necessarily accompanied by a stable AOB community, and provides insight into parameters controlling the AOB community dynamics within bioreactors with stable nitrification.

## Introduction

Ammonia in aquatic environments can be toxic to fish and other aquatic life and contributes to eutrophication of water bodies [1]. Accordingly, removal of ammonia in wastewater is one of the primary tasks of the modern wastewater treatment process.

A widely used method to remove ammonia in wastewater treatment plant (WWTP) is biological nitrification by which ammonia is oxidized to nitrite by ammonia oxidizing bacteria (AOB) and then nitrite is subsequently oxidized to nitrate by nitrite oxidizing bacteria (NOB). Although activated sludge is a common process for wastewater treatment, nitrification failure unfortunately occurs frequently in many WWTPs [1], [2], since nitrifiers, especially AOB, grow very slowly, and they are highly sensitive to several environmental and engineering factors, including temperature, pH, dissolved oxygen (DO), and a wide variety of chemical inhibitors [3], [4]. Therefore, a better understanding of the microbial ecology of AOB in WWTPs could potentially improve the nitrification stability [5].

Culture-dependent methods are biased by the selection of species which obviously do not represent the real dominance structure, and hence give a poor understanding of AOB community structure [6]. To overcome these limitations, currently molecular biology techniques can be used to analyze sequences of the 16S rRNA and *amoA* genes to reveal AOB communities in various environments [7].

A number of studies have used molecular biology techniques to examine the influence of various factors on AOB community structure in WWTPs [4], [8], [9], [10], [11]. To date, however, the relative influence of specific deterministic environmental factors to AOB community dynamics in WWTP (with associated concurrent changes in a multitude of environmental parameters) is uncertain [2].

Also, the ecological principles underlying AOB community dynamics and nitrification stability and how they are related are poorly understood. Wittebolle et al [12] have showed that in a laboratory-scale sequential batch reactor (SBR), the AOB community had a weekly change rate of 13±5% on 16S rRNA gene level despite the stable function of nitrification. This suggested that in laboratory-scale reactors, the functional stability of nitrification was not necessarily accompanied by AOB community stability. In the larger dimensional WWTPs, it remains unknown whether the frequent arrival of allochthonous organisms leads to a more stable or more dynamic community structure [8]. An equilibrium model based on island biogeography also predicts that the scale of the bioreactor will affect the microbial communities within it [13]. Wells *et al.* [2] observed the temporal oscillations of AOB populations within a full-scale WWTP while nitrification remained stable. However they did not evaluate the change rate, thus it is not clear whether the larger dimensional WWTP harbor a more stable or more dynamic community structure. Therefore, it is necessary to study the relationship between functional stability of nitrification and the AOB community dynamics in larger dimensional WWTPs.

The aim of this study was to determine if the functional stability of nitrification was correlated with a stable microbial community structure in a relatively large-scale WWTP (a pilot-scale plant with a volume of 72 m^3^), and to identify operational and environmental factors that most significantly correlate with the dynamics of AOB community structure. Accordingly, the samples were collected from a pilot-scale WWTP every 15 days over a one-year period, and then these samples were analyzed via *amoA*-based Terminal Restriction Fragment Length Polymorphism (T-RFLP) to monitor the AOB community dynamics, and used multivariate statistical tools to identify operational and environmental variables that significantly correlated to the dynamics of AOB community.

## Results

### System performance

**Figure 1:**
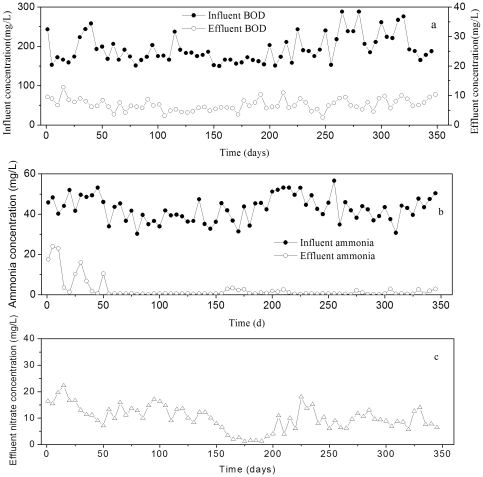
Functional performance in the pilot-scale wastewater treatment system over 345 days. (a) Influent and effluent BOD concentrations. (b) Influent and effluent ammonia concentrations. (c) Effluent nitrate concentrations.

The process performance of the pilot-scale WWTP during the study period is presented in Figures 1 and S1. Despite BOD in the influents varying from 153 to 288 mg/L, the BOD removal efficiency was always excellent (>92%) over the duration of the study. The average BOD concentration in the effluent was found to be below 10 mg/L (Figure 1 a). During most of the study period, the effluent ammonia concentrations were below 2 mg/L, except in the first 60 days (Figure. 1 b). The average nitrite concentration of final effluent was smaller than 1 mg/L and no nitrite accumulation phenomenon was detected in the final effluent. The average concentration of nitrate in the effluent was 26 mg/L (Figure 1 c). The conversion of ammonia to nitrite and further to nitrate was almost complete in the system over most of the study period.

### Temporal dynamics of AOB community

**Figure 2:**
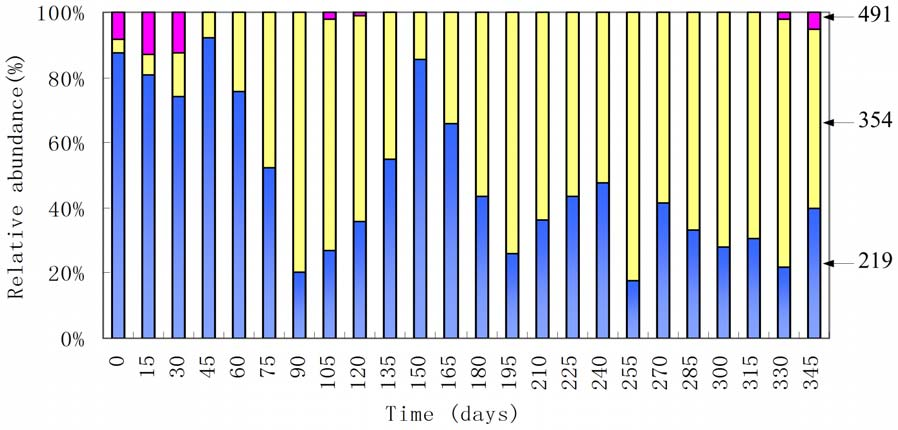
Histograms of T-RF relative abundances in the system for *Taq*I T-RFLP profiles. The relative abundance is the ratio of the peak area of a given T-RF in a given sample to the sum of all T-RFs in that sample expressed as a percentage. Arrows indicate the sizes of the restriction fragments for the abundant T-RFs in base pairs.

**Figure 3:**
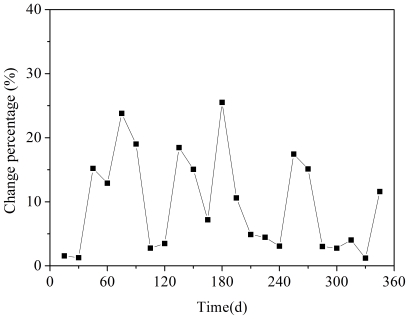
Moving-window analysis based on AOB T-RFLP profiles. Each data point is the change percentage between the bacterial communities of 2 consecutive dates. The time span between two consecutive dates is 15 days.

During the study period from April 2007 to March 2008, 24 activated sludge samples taken from the system were analyzed by T-RFLP of the *amoA* genes (Figure 2). The T-RFs with relative abundance below 2% were regarded as background noise and excluded from the analysis, and then, there were 3 remaining T-RFs: 219 354 and 491 bp. The total relative abundance of rare T-RFs (those with relative abundance below 2%) was not more than 5%. Moving-window analysis showed that change rates of AOB communities between two consecutive dates (15 d) were between 1% and 25% (Figure 3). The average change rate (except for the first 60 days) was 10%±8%. In particular, the relative abundances of the 219 and 354 bp T-RFs varied greatly. Prior to day 60, the 219-bp T-RF was present at a relative abundance of >70%, but from day 60–90, it decreased dramatically, reaching 20% on day 90. After day 90, its relative abundance increased gradually, reaching 87% at day 150. From day 180 to 345, the relative abundance of this T-RF dramatically fluctuated between 18%–47%. In contrast, the 354-bp T-RF with a relatively low ration of <15% before day 45, increased dramatically and reached 80% on day 90. After day 90, its relative abundance decreased gradually to 13% on day 150. After day 180, the relative abundance of this T-RF fluctuated between 53%–82%. The 491 bp T-RF can be only detected in a small number of samples (days 0, 15, 30, 105, 120, 330 and 345) with the relative abundance of <13%.

### Correlation of T-RFs and AOB clusters

**Figure 4:**
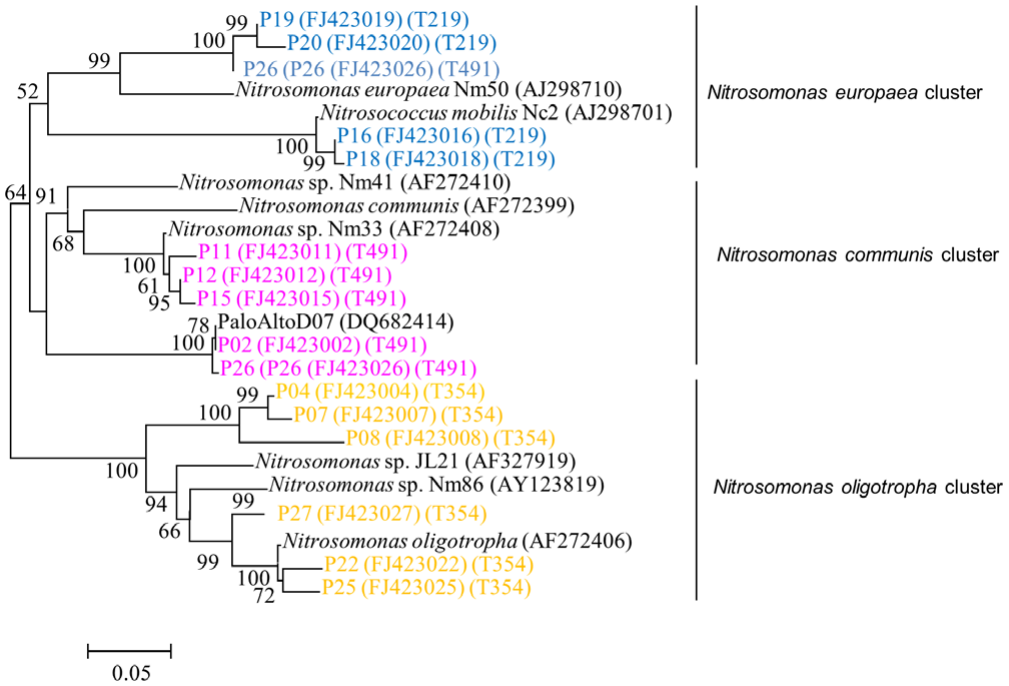
Phylogenetic tree showing the relationships of partial *amoA* gene sequences to reference sequences from the GenBank database. The tree was constructed with the neighbor-joining method using 450 nucleotide positions. Clone sequences are named beginning with “P”, and T-RF sizes are given in parentheses beginning with “T”.

Clone libraries were constructed for the sample collected on day 15. The selection of this specific sample allowed for the correlation of clone sequences with T-RFs that were particularly interesting in the T-RFLP analysis. 46 *amoA* clones were sequenced and then grouped on a 97% similarity criterion. One representative sequence from each group (a total of 15 sequences) was chosen for phylogenetic analysis. Phylogenetic analysis cloned *amoA* genes of each group showed that all of the sequences were closely related to *Nitrosomonas* spp. (Figure 4). All of the *amoA* clones were subjected to T-RFLP analysis. By combining the results of sequencing the *amoA* genes and the T-RFLP profiles of clones, it could be determined what AOB cluster each peak represents. The results revealed that, in the system studied, the 219, 354, and 491 bp peaks indicated members of the *Nitrosomonas europaea* cluster, *Nitrosomonas oligotropha* cluster and *Nitrosomonas communis* cluster respectively. On day 15, although a minor fraction (8%) of T-RFs was associated with T-RF 354 bp, the results were agreed with a number of previously published papers [1], [2], [14].

### Correlations of operational data and AOB community dynamics

**Figure 5:**
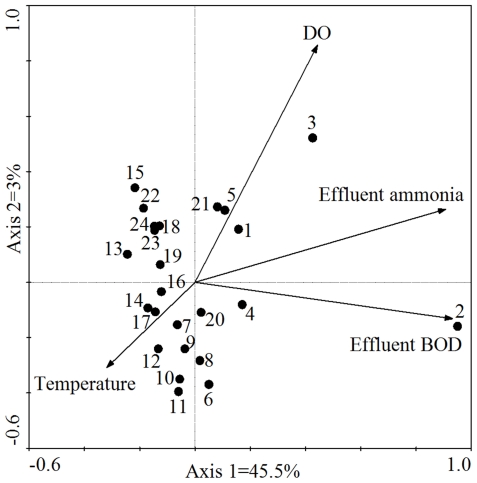
Canonical correspondence analysis (CCA) biplot based on T-RFLP data and measurable variables (operational and environmental) in the pilot-scale wastewater treatment plant. Arrows indicate the direction and magnitude of measurable variables associated with AOB community structures. Each Circle and associate number represents a different AOB community structure from a specific sampling date. Environmental variables were chosen based on significance (*P*<0.05) calculated from individual CCA results and variation inflation factors calculated during CCA.

Direct gradient analysis was widely used to study the correlations between microbial community structures and environmental variables. The two most commonly used direct ordination techniques are Redundancy Analysis (RDA) and Canonical Correspondence Analysis (CCA). RDA is the constrained form of Principle Component Analysis (PCA), and is inappropriate under the unimodal model. CCA is the constrained form of Correspondence Analysis (CA), and therefore is preferred for most ecological data sets (since unimodality is common). Therefore, we chose CCA to examine the relationships between AOB community structures and environmental variables. The results of CCA showed that the ordination explained all the variance (100%) of species–environment relations (i.e. T-RFs-environmental data), although this represented about a half of the variance of species data (i.e. T-RFs; 51%). Global Monte-Carlo permutation tests demonstrated that both the first axis and all axes combined explained a significant amount of the variability in AOB community structure (*P*<0.01). The first axis was positively correlated with effluent ammonia and BOD, but negatively correlated with temperature. The second axis was positively correlated with DO (Figure 5). The angle of an environmental parameter arrow in the ordination plot indicates how that variable is associated statistically with the major extracted axes, and the length of an arrow indicates the strength of the correlation with the axis. Of the **10** operational and environmental variables tested in this study, DO, temperature, effluent ammonia and BOD concentrations were significantly linked to the AOB community variability (*P*<0.05).

Mantel test was further performed to determine the most key individual environmental factors affecting the AOB community structure (Table 1). The results showed that DO temperature, effluent ammonia and BOD concentrations were significantly (*P*<0.05) correlated with the AOB community structures, which is in accordance with the results of CCA.

**Table 1:** Relationship of whole AOB community structure to individual environmental variables revealed by Mantel test.

Environmental variable	r_M_ a	*P*
Influent BOD	0.16	0.08
Effluent BOD	0.40	**0.01** b
Influent ammonia	0.12	0.08
Effluent ammonia	0.34	**0.01**
Effluent nitrite	−0.02	0.52
Effluent nitrate	0.14	0.06
Removal efficiency of ammonia	0.32	0.13
DO	0.13	**0.03**
Temperature	0.30	**0.01**
SVI	0.38	0.10
MLSS	−0.07	0.74
SRT	−0.01	0.49

a r_M_, Mantel's correlation coefficient.

b Boldface values indicate significant *P* values (<0.05).

## Discussion

### Correlation of T-RFs and AOB clusters

In this study, AOB community dynamics were examined by T-RFLP, a highly reproducible and robust technique often used to characterize microbial community structures in different habits [15]. For T-RFLP technique, an increasingly popular trend has been to associate T-RF with represent microorganisms by using in silico digestion or T-RFLP of clones. However, some previous works reported that there was a discrepancy ranging from 1 to 7 bp between in silico-determined T-RF length and actual T-RF length determined by clone T-RFLP, which can significantly affect identification of microbial species [16]. In this study clone T-RFLP, instead of in silico digestion, was performed to correlate representative species with particular T-RFs. Clone T-RFLP results revealed that, in the present study, the 219, 354, and 491 bp T-RFs indicated members of the *Nitrosomonas europaea* cluster, *Nitrosomonas oligotropha* cluster and *Nitrosomonas communis* cluster respectively, which are all related to *Nitrosomonas* spp., not to *Nitrosospira* spp. This result is consistent with most previous studies [8], [15], [17], [18], but is in contrast to a few studies which found *Nitrosospira* spp. to be the dominant AOB [19]. In pure-culture studies, fast-growing *Nitrosomonas*, such as *N. europaea*, can have a maximum specific growth rate (μ_max_) as high as 0.088/h whereas *Nitrosospira* sp. has a μ_max_ ranging from 0.033 to 0.035/h [1]. This growth advantage may favor *Nitrosomonas* over *Nitrosospira* as the prevailing species in activated sludge.

### AOB community dynamics

Analysis of the AOB T-RFLP profiles revealed that, during a period of stable nitrification, AOB communities were not stable, with an average change rate (every 15 days) of 10%±8%. Generally, the average change value Δ*_t_*_(7 days)_ below 10% was regarded as a low level dynamics (stable) and 10–30% was medium level dynamics [15]. Wittebolle *et al.* [12] found that in a sequential batch reactor (SBR) and a membrane bioreactor (MBR) with functional stability, the Δ*_t_*_(7 days)_ values based on DGGE profiles were 13%±5% and 25±14% for their respective AOB subgroups, which is higher than values observed in this study. A more stable AOB community structure in this study could be explained by the difference in scale between the 72 m^3^ pilot-scale plant used in this study and the 25-liter lab-scale reactor used by Wittebolle et al in 2008. An equilibrium model based on island biogeography predicts a more stable community structure in the larger dimensional systems [13]. Also, the different methodologies employed (DGGE versus T-RFLP) and the different genes (16S rRNA versus *amoA* genes) that were targeted by the two studies probably contribute to the difference of AOB change rates in the two reactors. In the previous study using the identical pilot-scale system to this study, it was found that the average change value Δ*_t_*_(15 days)_ of bacteria was 20%±11% during the period of stable function [20], which is higher than that of AOB in this research. The reason may be explained as that AOB have lower growth rate than the general bacteria in WWTPs.

In this study, our results demonstrate that the functional stability was not necessary accompanied by a stable microbial community. However, this does not mean that microbial community dynamics are irrelevant to functional stability. The dramatic changes of microbial community structure related to functional instability can and do occur. Gentile *et al.* [21] monitored the bacterial community dynamics in two dispersed-growth denitrifying reactors for about half a year, and found that during the period of functional instability, with high effluent nitrate concentrations, the community structure changed considerably, and the dynamics correlated significantly with effluent chemistry. Overall, the moderate dynamics of the microbial community, rather than dramatic or abrupt change, to adapt to changes in environments are important for the stable performance of the wastewater treatment systems [22]. Brionesa *et al.* [13] asserted that functional stability is not determined by microbial community structure, but of functional redundancy, which is ensured by the presence of a reservoir of species that can perform the same ecological function.

### Correlations of operational data and AOB community dynamics

Of the 10 operational and environmental variables tested in this study, DO emerged in CCA ordination and Mantel test as the important explanatory variable affecting the dynamics of AOB community. This agrees with the findings of Wells *et al.* [2], who suggested, based on T-RFLP surveys of AOB community dynamics of a municipal WWTP, that DO is one of the most influential variables on AOB community dynamics. Park *et al.* [14] compared the AOB community structures in two lab-scale bioreactors operated with high-DO (8.5 mg/L) and low-DO (0.24 and 0.12 mg/L) concentrations for a period of 300 days. The results showed that the AOB members present in the low-DO and high-DO bioreactors were both from *N. europaea* lineage, but phylogenetically different, indicating that DO exerts a significant selective pressure on AOB communities. However Park *et al.* [23] demonstrated that DO concentration did not influence the AOB community, but rather the activity of AOB. Similar results have also been obtained in several full-scale WWTPs in Tokyo by Limpiyakorn *et al.* [17]. Furthermore, Lydmark *et al.* [24] concluded that there was not yet enough data to allow correlations between AOB clusters and oxygen levels. Further research is needed to explain the effect of DO on the AOB community structure.

As with DO, effluent ammonia concentrations were strongly and significantly linked to AOB community dynamics. Ammonia concentration within bioreactor has previously been shown to be the important factor to shape the community structure of AOB. Lydmark *et al.* [24] found that ammonia concentration was an important structuring factor based on the DGGE analysis of the AOB community dynamics in four pilot-scale wastewater treatment systems with different ammonia concentration. In addition ammonia concentration, the ammonia load in the wastewater treatment system has also been demonstrated the significant influential factors on AOB communities by Limpiyakorn *et al.* [8].

In addition to DO, effluent ammonia, AOB community dynamics were also significantly linked to effluent BOD concentrations. The treatment process in this study is A^2^O, in which the heterotrophic bacteria and autotrophic AOB coexisted together. Although autotrophic AOB cannot use BOD as carbon source and electron donors, BOD is critical during the competition between heterotrophic bacteria and autotrophic AOB [25]. The competition may have effects on the AOB community structures with the bioreactor. In a compact suspended carrier biofilm reactor used to simultaneous nitrification and denitrification, Xia *et al.* [25] found that AOB and heterotrophic bacteria communities were influenced by the organic matter concentrations.

In addition to DO, effluent ammonia and BOD, AOB community dynamics were also significantly linked to the water temperature in the pilot-scale plant. Some previous studies have also demonstrated the importance of temperature. In a full-scale bioreactor treating saline wastewater, Park *et al.* [26] demonstrated that temperature was the most significant factor affecting the AOB community structure, rather than sodium, chloride and other environmental variables. In an aerobic biofilm reactor, Park *et al.* [27] found that low temperature could not only decrease the attached biomass and activity of AOB, but also produced a change in the composition of the AOB species, which resulted in the failure of nitrification. In contrast, Limpiyakorn *et al.* [17] used denaturing gradient gel electrophoresis (DGGE) to evaluate the effects of seasonal change on the AOB communities in 12 WWTPs, and the results showed that AOB communities of most systems were nearly identical regardless of the season change, indicating that temperature has no detectable effects on the AOB communities. Tourna *et al.* [28] also found that temperature had almost no effect on the AOB communities in agricultural soil based on the analysis of DGGE profiles of AOB across different incubation temperatures. Further research is needed to explain this discrepancy.

It should be noted that the correlation between ammonia removal efficiency and AOB community structure dynamics was not statistically significant (*P*>0.1). Part of the reason could be that ammonia removal efficiency is not only determined by microbial community structure, but also the quantity of AOB in WWTP. Further research is needed to relate the quantity of AOB to the ammonia removal efficiency in WWTP.

In CCA analysis of this study, the deterministic environmental and operational factors explained as much as 51% of the variance for the AOB community, thus 49% of the variance was determined by unknown factors. It is reasonable to expect that some additional factors, such as stochastic dispersal and immigration, predation and some unmonitored inhibitory chemicals may play an influential role in mediating AOB community dynamics in wastewater treatment systems.

### Conclusions

The AOB community dynamics in a pilot-scale WWTP were monitored by T-RFLP combined with clone library. The results showed that the AOB community change rate was 10%±8%, despite the stable nitrification. CCA and Mantel test was used to reveal relationships between the AOB community dynamics and operational and environmental parameters. The results revealed that AOB community dynamics correlated most strongly with DO, effluent ammonia, effluent BOD and temperature. The findings enrich the theory involving the relation between AOB community dynamics and stable nitrification, and offer insight into parameters controlling the AOB community dynamics in WWTPs.

## Methods

### The pilot-scale WWTP and sampling

The pilot-scale WWTP treating sewage wastewater in this research was operated with an anaerobic/anoxic/aerobic (A^2^O) process. The treatment capacity was 144 m^3^/d. The working volumes of the three compartments (anaerobic, anoxic and aerobic zones) in the system are 6, 12, and 54 m^3^ respectively. The influent rate was 6 m^3^/h, which gave a Hydraulic Retention Time (HRT) of 1, 2, and 9 h respectively in the three zones. In the study period, DO and pH were maintained at a relatively steady state, ranging from 1.8 to 2.0 mg/L and 7.0 to 7.2 respectively in the aerobic zone. The Mixed Liquor Suspended Solids (MLSS) were controlled at 4.5–6.0 g/L and the Solid Residence Time (SRT) was 7–10 d. The temperature range in summer and winter was 23–26°C, and 17–20°C respectively, and the average temperature in fall and spring was around 21°C.

MLSS samples were collected from the end part of the aeration tank every 15 days from April 2007 to March 2008. For archiving, each sample of 1.5 mL was dispensed into a 2 mL sterile Eppendorf tube and centrifuged at 14,000 *g* for 10 min. The supernatant was decanted, and the pellet was stored at −20°C prior to analysis.

### DNA extraction

The pellets of activated sludge samples were washed three times by centrifuging using sterile high-purify water for 5 min at 15,000 *g*. DNA extraction was then performed using a FastDNA® SPIN Kit for Soil Kit (MP Biotechnology, USA) according to the manufacturer's protocol.

### PCR amplification and purification

For clone library construction and sequencing, the *amoA* genes were amplified from the community DNA using the primers *amoA*-1F (5′-GGGGTTTCTACTGGTGGT-3′) and *amoA*-2R (5′-CCCCTCKGSAAAGCCTTCTTC-3′) [29]. For the T-RFLP analysis the same primer sequences were used, but *amoA*-1F was fluorescently labeled with the dye 5-carboxyfluorescein (FAM). PCR was performed according to the protocol of Wang et al. [4].

### Cloning and sequencing

Prior to cloning, the amplified unlabeled *amoA* gene fragments were purified using the QIAquick PCR Purification Kit (Qiagen, Germany). Purified PCR products were ligated into pGEM-T Easy cloning vectors (Promega, USA), and used to transform competent *Escherichia coli* DH5α cells (Tiangen, China) as described in the manufacturer's protocol. Transformants were selected by ampicillin resistance, and blue-white screening was performed to identify clones with inserts. Primers T7 and SP6 were used to perform colony PCR and to verify that the insert size was correct. Following PCR confirmation of the insert size, the amplified inserts were run on 2% (wt/vol) agarose gels. The samples containing inserts of the estimated size were used for subsequent sequencing. Sequencing was done by a commercial company (Nuosai gene, China).

Cloned *amoA* gene sequences and sequences of the closest BLAST matches were aligned with the software ClustalX 1.81, and a phylogenetic tree generated by the neighbor joining method using software Mega 4.0 (University College Dublin, Ireland) [30].

### T-RFLP analysis

Purified PCR products were digested with *Taq* I restriction endonuclease (TaKaRa, Japan) and then were used for capillary electrophoresis on an ABI PRISM 3130-Avant Genetic Analyzer (Applied Biosystems, USA) in GeneScan mode. The detailed procedures were presented in a previous paper [4]. To avoid detection of primers and uncertainties of size determination, terminal fragments smaller than 50 bp and larger than 500 bp were excluded from further analysis. The relative abundance of T-RFs within the sections was determined by calculating the ratio between the area of each peak and the total area of all peaks within one sample. The T-RFs with relative abundance below 2% were regarded as background noise and excluded from the analysis.

To identify which species the T-RF peaks represented, T-RFLP was performed using each *amoA* gene clone as a template DNA. The T-RFLP protocol was the same as that detailed above for community DNA. Because each clone contained one unique *amoA* gene fragment, each T-RFLP profile had only one peak (T-RF). By comparing the results of *amoA* gene sequencing and the T-RFLP profiles of clones, the species each T-RF represented could be determined.

### Statistical analyses

Moving-window analysis was used to characterize the change rate of AOB community in this study. Firstly, a matrix of similarities for each activated sludge T-RFLP profile was calculated based on Pearson product-moment correlation coefficients. Then, each similarity percentage value was subtracted from the 100% similarity value to get the change values. Finally, moving-window analysis was performed by plotting the change values between day *x* and day *x*-15 [31], [32]. The average change value Δ*_t_*_(15 days)_ was calculated as the average and standard deviation for the respective change values [33]. All statistical analyses were performed using SPSS 13.0 software (SPSS Inc., USA).

Canonical Correspondence Analysis (CCA) was used to reveal relationships between AOB community dynamics and operational and environmental parameters. The CCA method uses two datasets: in this study, the first set consisted of the T-RFLP patterns for each sample, and the second set consisted of the same day operational variables (temperature, DO, biomass, sludge retention time, influent and effluent BOD, influent and effluent ammonia, ammonia removal efficiency and effluent nitrate). CCA generates an ordination plot showing similarities of T-RFLP patterns among samples. In ordination plots, the gradients of explanatory variables are indicated by arrows. The length of an arrow indicates the relative importance of that parameter to the ordination. CCA analyses were performed focusing on interspecies distances using the software Canoco for Windows 4.5 (Biometris, The Netherlands). Statistically important explanatory variables were identified by the forward selection method using a Monte Carlo permutation test (499 permutations under the full model). Operational variables that failed to contribute significant improvement (P<0.05) to a model's explanatory power were excluded from final CCA analyses.

Mantel test was further performed to determine the most key individual environmental factors affecting the AOB community structure. Mantel test were performed using R 2.13.1(http://www.r-project.org). The Mantel test is a statistical test of the correlation between two matrixes and is commonly used in ecology [34].

## Supporting Information

Figure S1 **Operational parameters in the pilot-scale wastewater treatment system over 345 days.** (a) Water temperature and MLSS concentrations. (b) Dissolved oxygen and sludge volume index. (c) Sludge retention time.
(TIF)
