# Supplementary figures and images for: Ammonia Oxidizing Bacteria Community Dynamics in a Pilot-Scale Wastewater Treatment Plant
Source: PLoS One. 2012 Apr 27;7(4):e36272. doi: 10.1371/journal.pone.0036272 (PMC3338686; doi:10.1371/journal.pone.0036272)

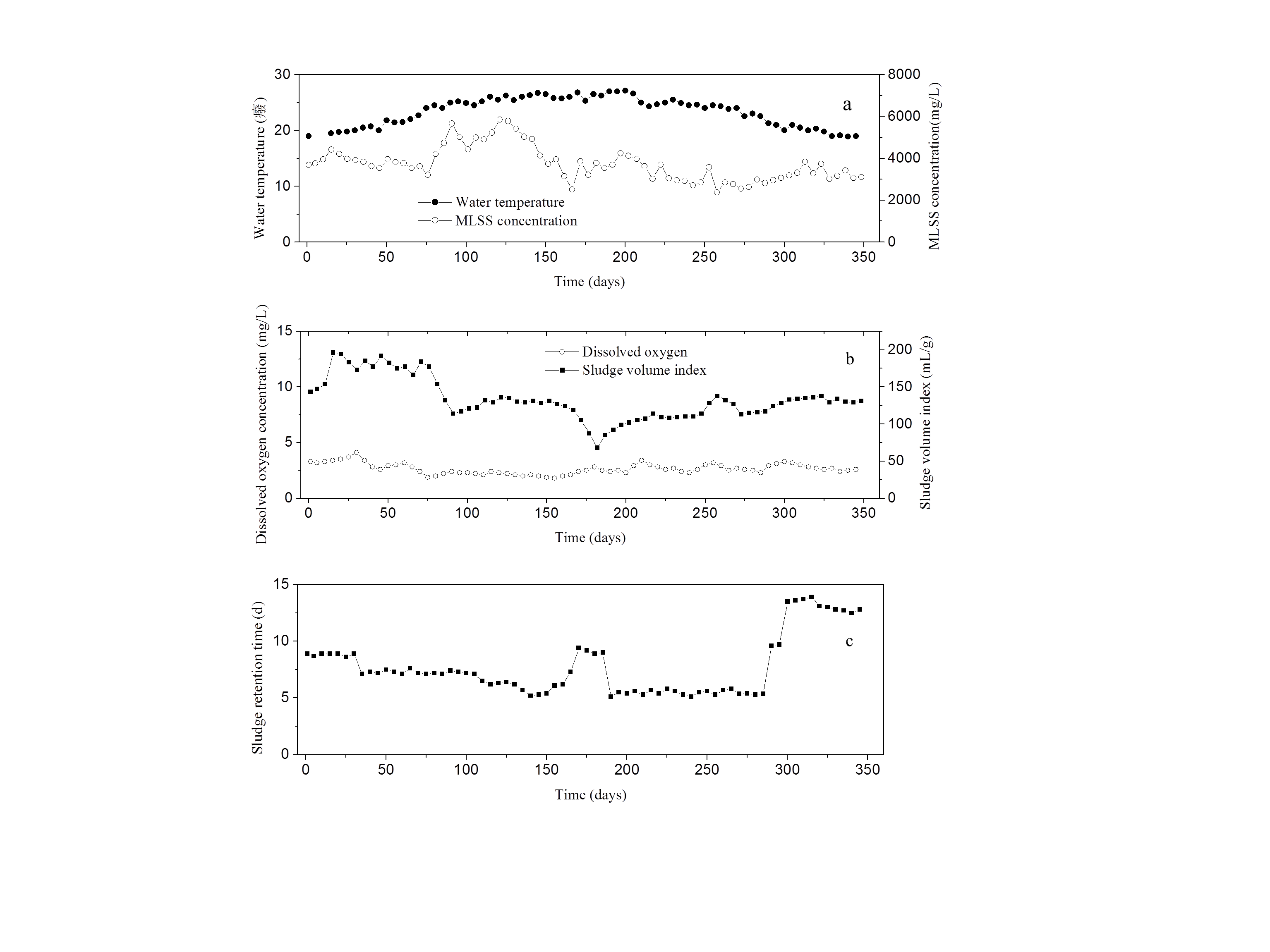

Supplement: Figure S1 — Operational parameters in the pilot-scale wastewater treatment system over 345 days. (a) Water temperature and MLSS concentrations. (b) Dissolved oxygen and sludge volume index. (c) Sludge retention time. (TIF) [file pone.0036272.s001.tif]
